# Effectiveness of Adapted Taekwondo, Multi-Component Training and Walking Exercise on Health Status in Independent Older Women: Study Protocol for a Randomized Controlled Trial (TKD & Aging Project)

**DOI:** 10.3390/biology11060816

**Published:** 2022-05-26

**Authors:** Pablo Valdés-Badilla, Tomás Herrera-Valenzuela, Eduardo Guzmán-Muñoz, Braulio Henrique Magnani Branco, José Zapata-Bastias, Boris Lucero, Franklin Castillo-Retamal

**Affiliations:** 1 Department of Physical Activity Sciences, Faculty of Education Sciences, Universidad Católica del Maule, Talca 3530000, Chile; fcastillo@ucm.cl; 2 Carrera de Entrenador Deportivo, Escuela de Educación, Universidad Viña del Mar, Viña del Mar 2520000, Chile; jzapata@uvm.cl; 3 Department of Physical Activity, Sports and Health Sciences, Faculty of Medical Sciences, Universidad de Santiago de Chile (USACH), Santiago 8370003, Chile; tomas.herrera@usach.cl; 4 Escuela de Kinesiología, Facultad de Salud, Universidad Santo Tomás, Talca 110231, Chile; eguzmanm@santotomas.cl; 5 Graduate Program in Health Promotion, Cesumar University (UniCesumar), Maringá 87050-900, Brazil; braulio.branco@unicesumar.edu.br; 6 The Neuropsychology and Cognitive Neurosciences Research Center (CINPSI Neurocog), Faculty of Health Sciences, Universidad Católica del Maule, Talca 3530000, Chile; blucero@ucm.cl

**Keywords:** public health, physical activity, older adults, active aging, combat sports

## Abstract

**Simple Summary:**

The benefits of regular physical activity on various health variables in older people have been extensively reported in scientific evidence. Resistance training, multi-component training, and walking exercise are the most commonly used physical activity interventions in older people, while interventions with adapted taekwondo are less common but have similar results to the strategies mentioned above. As a result, this study protocol aims to analyze and compare the effects of an adapted taekwondo program with respect to multi-component training and walking exercise on health status in independent older women. Secondarily, we analyze the variability of the inter-individual response and compare it according to the designated training system. According to the current scientific evidence we expect that adapted taekwondo will produce more significant effects and greater inter-individual responses in cognitive status, brain activity, health-related quality of life, and postural balance than the other training methods. If this intervention proves effective, it could be an alternative for older women.

**Abstract:**

This study protocol aims to analyze and compare the effects of an adapted taekwondo program with respect to multi-component training and walking exercise on health status in independent older women. Secondarily, we analyze the variability of the inter-individual response and compare it according to the designated training system. The sample will consist of 64 women between 60 and 65 years, randomly assigned to experimental group 1 (*n* = 16; adapted taekwondo), experimental group 2 (*n* = 16; multi-component training), experimental group 3 (*n* = 16, walking exercise) or control group (*n* = 16; no intervention). The experimental groups will perform the designated training for three sessions (60 min per session) per week over 16-weeks, while the control group will not receive any treatment. The main outcome will provide information about (i) blood pressure, (ii) lipid profile, (iii) frequency of food consumption, (iv) body composition, (v) cognitive status, (vi) brain activity, (vii) health-related quality of life (HRQoL) and (viii) physical-functional fitness. Our hypothesis indicates that adapted taekwondo produces more significant effects and greater inter-individual responses in cognitive status, brain activity, HRQoL, and postural balance than the others training methods. If this intervention proves effective, it could be an alternative for older women.

## 1. Introduction

Scientific evidence has extensively documented the benefits of regular physical activity on various health variables in older people [1,2,3,4,5]. For example, a significant reduction in resting blood pressure and heart rate [4], lower incidence of cardiovascular events [6] and falls [1,2], a significant increase in cardiorespiratory fitness, muscular endurance and strength, flexibility, agility, and dynamic balance [7,8], as well as significant improvements in attention [9] and cognitive processing speed [10]. Facts that, as a whole, favor functional independence and improve health-related quality of life in older people [3,4].

Some of the most recurrent physical activity strategies to intervene in older people correspond to resistance training [1,5], multi-component training [7,11], and walking exercise [6,12], probably because they have greater diffusion, scientific support, and versatility to dose the magnitude of the load and the exercises to the characteristics of the participants [5,13]. Following the above, government-sponsored physical activity programs for older people have reported endurance, strength, flexibility, balance, agility, walking, healthy dancing, and recreational sports as the main activities. However, their average adherence rate reaches 50% [4].

In the same context, physical activity interventions for older people based on Olympic combat sports have reported a mean adherence greater than 80% [8,13,14], with similar physical, physiological, and psychoemotional outcomes to other physical activity intervention strategies such as those mentioned above (resistance training, multi-component training, and walking exercise). Specifically, interventions with adapted taekwondo in older people have reported a significant increase in muscle strength of the lower [9,15] and upper limbs [15], cardiorespiratory fitness [9], flexibility [9], agility and dynamic balance [9,15,16,17], as well as significant improvements in the multidirectional reach test and a significant decrease in the gait stability ratio [17]. Cho and Roh [9] reported a significant increase in brain-derived neurotrophic factor, vascular endothelial growth factor, and insulin-like growth factor-1, while Lee, Scott, Pekas, Lee, Lee and Park [15] reported a significant decrease in resting epinephrine. In summary, taekwondo improves the health status of older people with adherence rates greater than 90% through interventions lasting 11 to 16 weeks [9,15,16,17].

On the other hand, in Chile, more than 70% of people over the age of 60 years old are classified as physically inactive [18], with women having the highest prevalence at 74.2%, and men at 54.7% [18]. Additionally, Chilean women are more sedentary (90%) and have a higher prevalence of overweight/obesity (74.8%) than men, who reach 83.3% and 73.6% for the same variables, respectively [19]. These data represent elements that would have a negative impact on the health status of Chilean older women [20]. Therefore, this study protocol aims to analyze and compare the effects of an adapted taekwondo program with respect to multi-component training and walking exercise on health status (blood pressure, lipid profile, frequency of food consumption, body composition, cognitive status, brain activity, health-related quality of life and physical-functional fitness) in independent older women. Secondarily, analyze the variability of the inter-individual response of the participants and compare according to the designated training system.

Based on previous studies [6,7,8,9,11,12,14,15,16,17] we hypothesized that: (i) adapted taekwondo produces significantly greater effects on cognitive status, brain activity, health-related quality of life, and postural balance than a multi-component training and walking exercise, and; (ii) older women who participate in the adapted taekwondo program have more favorable inter-individual responses (higher percentage of responders) in cognitive status, brain activity, health-related quality of life and postural balance compared to women who participate in a multi-component training and walking exercise.

## 2. Material and Methods

### 2.1. Study Design

The study includes an experimental design (randomized controlled trial), double-blind, repeated measures, four parallel groups (three interventions and one control), and a quantitative approach. The methodology followed will be the Consolidated Standards of Reporting Trials Statement (CONSORT) methodology [21].

### 2.2. Ethical Approval

The current protocol has been reviewed and approved by the Scientific Ethics Committee of the Universidad Católica del Maule, Chile (approval number: N°29-2022) and developed following the Declaration of Helsinki for work with human beings. In addition, it has been registered in the Clinical Trial Protocol Registry and Results System (ClinicalTrials.gov) of the United States of America (code: NCT05275140; https://clinicaltrials.gov/ct2/home, accessed on 22 April 2022).

### 2.3. Sample Size Calculation

The sample size calculation indicates that the ideal number of participants per group is 16. According to previous studies [22], for this calculation, an average difference of 3.46 repetitions (chair stand test) was used as the minimum difference required for substantial clinical relevance, with a standard deviation of 3.38 repeats, considering an alpha level of 0.05 with 90% power and an expected loss of 15%. GPower software (Version 3.1.9.6, Franz Faul, Universiät Kiel, Kiel, Germany) will be used to calculate statistical power.

### 2.4. Randomization and Blinding

Sixty-four women over 60 years of age will be invited to participate in the study voluntarily and then will be electronically randomized [23] and assigned to either experimental group 1 (*n* = 16; adapted taekwondo: TKD), experimental group 2 (*n* = 16; multi-component training: MCT), experimental group 3 (*n* = 16, walking exercise: WE) or the control group (CG, *n* = 16; no intervention).

### 2.5. Participants

**Figure 1:**
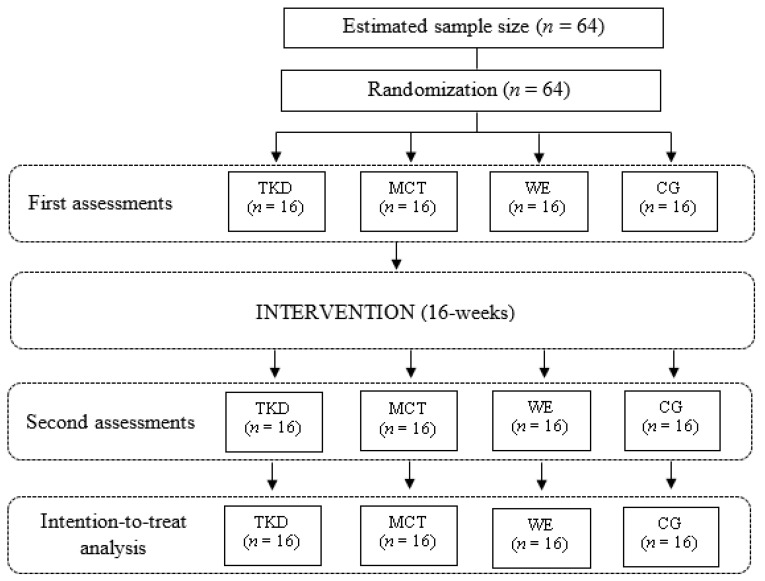
Study flowchart. Legends: CG: Control group (no intervention). MCT: multi-component training. *n*: number of participants. TKD: Adapted taekwondo. WE: Walking exercise.

The participants will be recruited in the city of Talca (Maule region), Chile. The inclusion criteria for the sample will be: (i) older women aged between 60 and 65 years old; (ii) presenting the ability to understand and follow instructions in a contextualized way through simple commands; (iii) independent, that is, have a score equal to or greater than 43 points in the Preventive Medicine Exam for the Older People (in Spanish, EMPAM) of the Ministry of Health of Chile [24]; and (iv) complying with at least 85% attendance at the sessions scheduled for interventions. Regarding the exclusion criteria, the following will be considered: (i) having any disabling disease; (ii) those women who have musculoskeletal injuries or who are undergoing physical rehabilitation treatment that prevents their typical physical performance; and (iii) those who are permanently or temporarily unable to engage in physical activity. As depicted in Figure 1, all those who meet the inclusion criteria will be included in the study.

### 2.6. Intervention

The TKD and MCT protocols will be distributed in three weekly sessions of 60 min on alternate days for 16-weeks (48 sessions). The general structure of the TKD and MCT protocols will include a 10 min warm-up consisting of joint mobility exercises and low-intensity aerobic work; then, for 40 min, the central part (TKD or MCT) will be developed to finish with a 10 min cool down through dynamic and static flexibility exercises. For their part, the WE protocol will be distributed in three weekly sessions of 45 to 60 min every other day for 16-weeks (48 sessions). A summary of the progression of all interventions is presented in Table 1.

#### 2.6.1. Adapted Taekwondo (TKD) Program

The main part of the TKD program will consist of non-contact activities, distributed in 10 min of basic postures and specific movements with the upper limbs (strikes and blocks) and 20 min of lower limb movements (displacement, postures, kicks) performed individually and in pairs with and without the implementation of taekwondo (shields and impact pads). In addition, choreographies or poomsae (sequence of arm and leg movements that simulate imaginary combat) specific to this modality were adapted to the characteristics of older women for 10 min. The training volume will be quantified by sets and the number of repetitions of the specific movements (i.e., strikes, blocks, displacement, postures, and kicks) with the rest of 2 min between sets. The intensity of the training takes into account the recommendations of previous studies with older people [9,15], so it will remain moderate to vigorous using the percentage of the maximum heart rate (HRmax) of each participant for its control (between 50% and 70% of the HRmax) with a heart rate sensor strap (H10, Polar Electro Oy, Kempele, Finland), which will be live transmitted throughout the protocols (TKD, MCT, and WE) through Bluetooth to a tablet (iPad 4, Apple, Inc., Cupertino, CA, USA) where the participants will be continuously monitored using the Polar Team app version 1.3 (Polar Electro Oy, Kempele, Finland). The rating of perceived exertion will be measured with the Borg scale (maximum value of 10 points). The adapted taekwondo sessions will be led by a Ph.D. student in health and certified as a taekwondo instructor by the National Sports Federation of Taekwondo WT. The sessions will take place in the multipurpose room of gym B of the Universidad Católica del Maule, which has an EVA rubber floor (tatami).

#### 2.6.2. Multi-Component Training (MCT) Program

The main part of the MCT will consist of 40 min of distributed work in a circuit, including resistance training focused on the large muscles of the upper limbs (i.e., biceps, triceps, deltoids, and latissimus dorsi) and lower limbs (i.e., quadriceps, hamstrings, glutes, and gastrocnemius) combined with exercises aimed at cardiorespiratory fitness, agility and postural control (static and dynamic balance), using elastic bands, poles, 2-kg medicine balls, and chairs, following previous recommendations [4,5,7,11,22]. The training volume will start (the first 4-weeks) with 3 sets of 10 repetitions per muscular exercise with a 2 min rest period between the sets, performing slow movements of two seconds in concentric contraction and four seconds in eccentric contraction [25]. Between weeks 5 to 8, the volume will increase to 4 sets of 10 repetitions per muscular exercise with 2 min of rest between sets. Between weeks 9 and 12, the sets will remain at 4, and the number of repetitions will be increased to 12; finally, between weeks 13 and 16, the number of sets and repetitions (4 × 12) will be maintained reducing the rest time to 90 s. The resistance training intensity will be moderate to vigorous (between 5 to 8 points), controlled with the OMNI-Resistance Exercise Scale of perceived exertion [26]. The intensity of the cardiorespiratory fitness, agility, and postural control will be maintained at moderate to vigorous (between 50% and 70% of the HRmax), controlled with a heart rate sensor strap (H10, Polar Electro Oy, Kempele, Finland), which will be live transmitted through Bluetooth to a tablet (iPad 4, Apple, Inc., Cupertino, CA, USA), and individualized according to the initial assessments of the participants and following previous recommendations [3,5]. Furthermore, the rating of perceived exertion will be used (Borg scale of 10 points). The MCT sessions will be directed by a Master’s student in physical activity sciences with experience working with older people. The sessions will take place in the multipurpose room of gym B of the Universidad Católica del Maule, which has an EVA rubber floor (tatami).

#### 2.6.3. Walking Exercise (WE) Program

The general structure of the WE program will include a 5 min warm-up consisting of joint mobility and flexibility exercises. Next, the main part will be developed for 30 to 45 min (increased by 5 min every 4-weeks), consisting of walking on flat ground, touring the university’s jogging circuit, and ending with 5 min cool down through dynamic and static flexibility exercises. The training volume will be regulated by the distance traveled using triaxial accelerometers (ActiGraph GT9X, Pensacola, FL, USA), and the intensity will be kept at moderate to vigorous (between 50% and 70% of the HRmax) controlled with a heart rate sensor strap (H10, Polar Electro Oy, Kempele, Finland), which will be live transmitted through Bluetooth to a tablet (iPad 4, Apple, Inc., Cupertino, CA, USA). The progression of the load will be adjusted every 4-weeks, increasing the walk by 5 min and regulating the cadence individually, which will increase the distance covered by the participants—the rating of perceived exertion (Borg scale of 10 points). The design of the WE considers recommendations and previous studies [3,6,12]. A Master’s student in physical activity sciences with experience working with older people will lead the walking sessions.

#### 2.6.4. Control Group (CG)

Regarding the CG, the individuals will participate in the assessments (initial and final) and will be asked to maintain their usual activities of daily living. A research assistant will contact the participants by telephone (once a week) to inquire about their health status and monitor their activities. At the end of the intervention period, the CG will be invited to participate in a physical activity program developed at the University based on the results of this study.

### 2.7. Outcomes and Procedures

**Figure 2:**
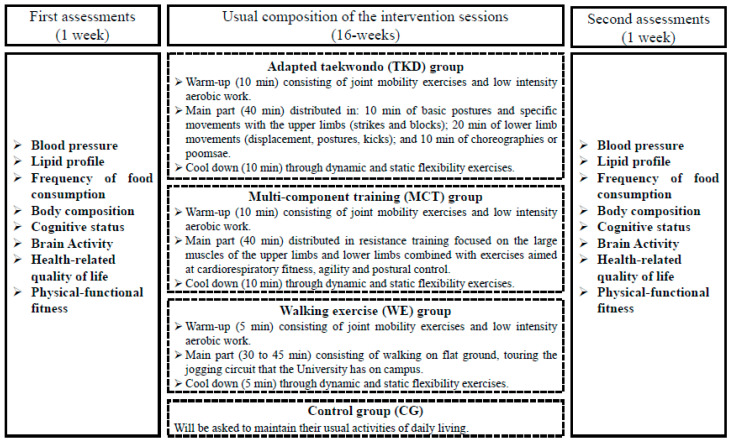
Assessments and usual sessions of the intervention. Legends: CG: Control group. MCT: Multi-component training. TKD: Adapted taekwondo. WE: Walking exercise.

The first meeting with the participants will be held to explain the scope and aim of the study, obtain the signature of informed consent, administer the anamnesis, and randomly assign them to the intervention groups. Next, for a week, the older women will be assessed on the variables considered for the study; later, they will participate in the designated training programs. After 16-weeks of intervention, older women will undergo the same initial assessments for one week. One of the researchers will supervise all the assessments, which will be carried out by an anthropometrist (body composition), a nurse (blood pressure, lipid profile, and frequency of food consumption), a speech-language pathologist (cognitive status and health-related quality of life), a psychologist specializing in neuroscience (brain activity), and two physical education teachers (physical-functional fitness). The evaluators will not have information about the training groups, nor will the participants know their identities (i.e., a double-blind shield). Figure 2 shows a summary of the assessments and sessions that are usually part of the intervention.

#### 2.7.1. Primary Outcomes

Blood pressure: An automatic pressure monitor will obtain the systolic and diastolic blood pressure (08A, CONTEC, Alsdorf, Germany). The participants will be evaluated based on at least 10 min of prior rest in a sitting position, with the back and arms supported and the legs without crossing, emptying the urinary bladder if necessary. In addition, women will be asked not to practice exercise, smoking, or drinking alcohol or coffee at least 30 min before the measurements. The first assessment will be taken in both arms to identify the arm with the highest blood pressure, and then two assessments will be carried out in the arm with the highest blood pressure (usually the dominant one), adding a third assessment if the difference between the measurements is greater than 5 mmHg.

Lipid profile: This will be determined in a fasting condition of at least 12 h; the extraction of 5 mL of capillary blood will be carried out by a nurse considering the necessary safeguards in the Human Performance Laboratory with the use of a meter (Cardiochek PA, USA) to determine total cholesterol, HDL cholesterol, LDL cholesterol, triglycerides, cholesterol without HDL, and total cholesterol/HDL cholesterol ratio.

Frequency of food consumption: The nutritional assessment will be carried out using the survey on eating habits, modified for use in older people, and validated by the Delphi method based on the judgment of 25 experts in nutrition [27]. The survey aims to measure the eating habits of older people and is made up of two areas of self-application. The first is composed of 12 items with a minimum score of 1 and a maximum score of 5 per question (Likert scale), which indicates the frequency of healthy habits, such as the frequency of consumption of recommended food groups, ranging from not consuming (1 point) up to the suggested day/week servings (5 points), with a score of the responses that varies from 12 to 60 points (higher value, better-eating habits) [27]. The second area consists of 7 items, foods or food groups identified as promoters of chronic non-communicable diseases (sugary drinks, alcohol, fried foods, fast food, sweet snacks, coffee), and a negative eating habit is added, such as adding salt to meals without tasting them; six of the questions have an identical score to the previous one (1, does not consume, to 5, more than three servings per day/week) and only one is rated from 1 to 3 (salt), reaching a value that ranges from 7 to 33 points (higher values indicate worse eating habits) [27].

Body composition: The following anthropometric measurements will be obtained: (i) body weight in kg with a digital scale (Seca 769, Germany; Accuracy of 0.1 kg); (ii) bipedal height in cm with a stadiometer (Seca 220, Germany; Accuracy of 0.1 cm); (iii) diameters (biacromial, transverse thorax, anteroposterior thorax, bi-iliocrestid, humeral, femoral) in cm with an anthropometer (Rosscraft Campbell 20; Accuracy of 0.1 mm); (iv) perimeters (head, arm relaxed, arm flexed in tension, forearm maximum, mesosternal thorax, minimum waist, maximum hip, maximum thigh, medial thigh, maximum calf) in cm using an inextensible measuring tape (Seca 201, Germany; Accuracy of 0.1 cm); and (v) skinfolds (tricipital, subscapular, supraspinal, abdominal, medial thigh, calf) in mm with a caliper (Harpenden, England; Accuracy of 0.2 mm). According to the International Society for the Advancement of Kinanthropometry (ISAK) [28], all assessments will be performed by an ISAK level II anthropometrist. Moreover, each woman’s body mass index will be calculated by dividing the body weight in kg by the bipedal height in m^2^. In addition, the percentage of fat mass and fat-free mass will be obtained using eight electrode tetrapolar bioimpedance (InBody 570^®^, Body Composition Analyzers, Seoul, Korea).

Cognitive status: This will be evaluated using the memory, phonetic fluency, and temporal-spatial orientation survey (in Spanish, MEFO) [29], which aims to determine cognitive impairment in older people. This survey classifies the participants into three levels: without cognitive impairment, with mild cognitive impairment, or with cognitive impairment. The advantage of the MEFO is that the level of schooling does not have an influence; its application is simple and brief [29]. The MEFO assesses deferred free recall, phonetic fluency with the letter P, and temporal-spatial orientation; furthermore, it has high sensitivity in the fields described and is validated for use with older people in Chile [29].

Brain activity: This will be recorded through a 64+8 channel surface electroencephalography (EEG) system (active-Two from BioSemi, Amsterdam, The Netherlands) using a mapping task and a reaction time task to measure spatial and visual-motor skills, in addition to the planning index of the participants, following previous recommendations [30].

Health-related quality of life: This will be obtained using the Health Survey Short Form (SF-36) version 2, which measures the attributes of eight health dimensions: physical function, physical role, body pain, general health, vitality, social function, emotional role, and mental health [31]. Each dimension is made up of a series of questions that together give a scale ranging from 0 (the worst health status for that dimension) to 100 (the best health status) [31].

Physical-functional fitness: This will be evaluated with the Senior Fitness Test, which allows evaluation of great reliability and easy application [32]. The order of application of the tests contemplated in the battery will be a chair stand test to assess the strength of the lower limbs, counting the number of repetitions made in 30 s. Using a 3-lb dumbbell and counting the number of repetitions made in 30 s, the arm curl test will assess upper-limb strength. A 2 min step test to assess cardiorespiratory fitness, recording the number of knee raises that reach at least an angle of 70° on the hip joint of each participant. The Chair sit-and-reach test to assess the flexibility of the lower limbs, measured in cm. The back scratch test to assess flexibility on the upper limbs, measured in cm. The timed up-and-go test to assess agility and dynamic balance, surrounding a cone at 8-ft (2.44 m) and recording time in seconds.

Following previous recommendations [33], the handgrip strength will be measured with a hydraulic dynamometer (Camry, model EH101, China). The participants will be located in a seated posture, aligned spine, shoulders adducted and without rotation, elbow flexed 90° to one side of the body, forearm, and wrist in a neutral position. The size of the hand will be considered to adjust the dynamometer, allowing a comfortable and functional grip of the instrument with an adequate closure of the metacarpal phalangeal and interphalangeal joints in the position of the fist, favoring contact between the first phalanx of the index and the thumb. Three attempts will be made for each hand, using the maximum value of the three registers. The postural balance will be obtained with a force platform (ArtOficio Ltd., Valparaíso, Chile) following previous recommendations [34]. The data will be acquired with a sampling rate of 40 Hz. The measurement of the postural balance will be carried out in a situation with open and closed eyes; each will have a duration of 30 s. Participants will be instructed to maintain the bipedal position as still as possible, with the arms relaxed at the side of the torso and the feet aligned with a separation similar to the shoulders’ width. The area and velocity variables of the center of pressure will be calculated using Matlab r2012a software (Mathworks Inc., Natick, MA, USA).

#### 2.7.2. Secondary Outcomes

Sociodemographic assessments: Age (years), academic level (primary, secondary, bachelor, master, Ph.D.), and civil status (married, separated, widowed, single, others).

### 2.8. Statistical Analysis

The Statistical Package for Social Science (SPSS) 25.0 will be used. Descriptive statistics will be performed where the summary and dispersion measures of the data will be calculated. The variables will be subjected to the Shapiro-Wilk tests for normality and Levene’s homogeneity of variance.

#### 2.8.1. Intention-to-Treat Analysis

A two-factor repeated measures ANOVA will be used to determine the effects of the intervention on the outcomes (group × time). Post-hoc tests will be performed with an alpha adjusted by Bonferroni to identify statistically significant differences. The effect sizes will be calculated utilizing Cohen’s d [35] within the group and between the groups, using the following equation: effect sizes = (post mean − pre mean)/standard deviation combined. The level of significance will be set at 5%. The intraclass correlation coefficient will be used to verify the measurement’s reliability, with a predetermined threshold of 0.80 being used to include the data in the analyses. The inter-individual variability of the intervention will be determined using previously reported criteria, and responders and non-responders will be classified using the technical error (TE) of measurement. This TE will be calculated according to the formula: TE = SD (standard deviation) dif/√2, where SD is the variance of the differences in the observed values of two repeated measurements in each test [36]. A non-responder is a person who does not show an increase or decrease (in favor of beneficial changes) in the study variable greater than twice the TE, far from zero. Thus, a shift in favor of beneficial health effects, which is <2 TE means the existence of a high probability (12 to 1 odds ratio) that the response to exercise is true [36,37]. In addition, Fisher’s exact test will compare groups of participants who were at 2 × TE calculated on each outcome as non-responders and more than twice the TE as responders [37].

#### 2.8.2. Analysis by Protocol

Only those volunteers who have completed more than 85% of the training sessions will be considered for this analysis. The same procedures as those indicated for intention-to-treat analysis will be conducted.

## 3. Discussion

Despite being considered a risky activity [38], TKD has recently reported favorable physical, physiological, and psychoemotional responses in older people [9,15,16,17]. Previous studies have found that after 12-weeks of TKD and WE, participants had a considerably better response on static balance than a control group, with no differences between TKD and WE [39]. While one case study found that a TKD intervention reduced the chance of falls and the fear of falling in a 97-year-old man, the author adds that the evidence on the benefit proportionality of TKD in comparison to regularly used physiotherapy therapies is still unclear [40]. In this regard, conducting a study comparing the group and inter-individual responses (responders vs. non-responders) in older women subjected to a TKD program to other physical activity strategies adapted to the characteristics of older people (e.g., MCT and WE) could support the use of TKD as an efficient and safe activity for older women without putting their health at risk.

On the other hand, it is typical for interventions with young taekwondo practitioners to be of short duration (i.e., 4-weeks) using high-intensity interval training with specific techniques and an emphasis on improving physical performance [41,42,43,44]. These interventions cannot be extrapolated to older people, since the international physical activity recommendations suggest regular moderate to vigorous-intensity activities with emphasis on resistance training [3,5], which favors functional independence [4]. In addition, young taekwondo practitioners are generally athletes of regional or national level [41,42,43,44]. Therefore, the aim of the interventions does not seek to favor the health of the participants but rather the sports performance, instead, the older people generally practice physical activity to improve their health status [4,22].

Following the above, proposing alternative physical activity strategies such as TKD programs for older people, especially for Chilean older women who have high rates of a sedentary lifestyle and overweight/obesity [18,19], can be a viable alternative for at least four reasons: (i) little space is required, which is consistent with the characteristics of Family Health Centers (in Spanish, CESFAM) and Community centers that host physical activity governmental workshops in Chile [22]; (ii) it requires tiny implementation because a large part of the activities are developed individually and in pairs [8,14]; (iii) low costs required to carry out this type of intervention, which would allow its use in a large population of older people [8]; and (iv) the variety of technical foundations that allow the use of innumerable combinations of movements, a fact that could generate greater motivation and adherence to training [8,14].

Among the possible limitations of the study protocol are: (i) difficulty recruiting older women who adhere to the study for 16-weeks plus the pre-intervention and post-intervention assessment periods; (ii) the impossibility of some participants to complete the sessions contemplated in the intervention due to the COVID-19 infection. Notwithstanding the above, our study will follow Chilean government guidelines for the safe return to the practice of physical activity in the context of the COVID-19 pandemic.

## 4. Conclusions

This study protocol will analyze and compare the effects of a TKD program with respect to MCT and WE on health status in independent older women. Secondly, it will analyze the variability of the inter-individual response of the participants and compare them according to the designated training system. Our hypothesis indicates that TKD produces more significant effects and greater inter-individual responses (higher percentage of responders) in cognitive status, brain activity, health-related quality of life, and postural balance than MCT and WE. If this intervention proves to be beneficial, it might be included in public and private physical activity programs for older women.

## Figures and Tables

**Table 1:** Progression for intervention (Training volume and intensity).

Program	Month	Weeks	Frequency (Weekly)	Total Time Per Session (min)	Exercise	Set	Rep	Rest	Intensity
TKD	1	1–4	3	60	UL	3	8	2 min	50–70% HRmax
					LL				
					Poomsae	---	6		
	2	5–8			UL	4	8		
					LL				
					Poomsae	---	6		
	3	9–12			UL	4	12		
					LL				
					Poomsae	---	6		
	4	13–16			UL	4	12	90 s	
					LL				
					Poomsae	---	6		
MCT	1	1–4		60	RT	3	10	2 min	OMNI-RES (5–8 points)
					CF				50–70% HRmax
					APC				
	2	5–8			RT	4	10		OMNI-RES (5–8 points)
					CF				50–70% HRmax
					APC				
	3	9–12			RT	4	12		OMNI-RES (5–8 points)
					CF				50–70% HRmax
					APC				
	4	13–16			RT			90 s	OMNI-RES (5–8 points)
					CF				50–70% HRmax
					APC				
WE	1	1–4		45	---	---	---	---	50–70% HRmax
	2	5–8		50	---	---	---	----	
	3	9–12		55	---	---	---	----	
	4	13–16		60	---	---	---	----	
Control group	1–4	16	Will be asked to maintain their usual activities of daily living.						

APC: Agility and postural control. CF: Cardiorespiratory fitness. HRmax: Maximum heart rate. LL: Lower limbs. MCT: Multi-component training. OMNI-RES: OMNI-Resistance Exercise Scale of perceived exertion. Poomsae: Sequence of arm and leg movements that simulate imaginary combat. Rep: Repetitions. RT: Resistance training. TKD: Adapted taekwondo. UL: Upper limbs. WE: Walking exercise.

## Data Availability

Not applicable.
